# Supplementary material for: Scaling the tail beat frequency and swimming speed in underwater undulatory swimming
Source: Nat Commun. 2023 Sep 9;14:5569. doi: 10.1038/s41467-023-41368-6 (PMC10492801; doi:10.1038/s41467-023-41368-6)
Supplement: Supplementary file 2 — Description of Additional Supplementary Files [file 41467_2023_41368_MOESM2_ESM.pdf]

Supplementary Data 1

The database we constructed and used in the article.

Supplementary Code 1

The Mathematica code and files used for determining the boundaries in the L-f plane.

Length(m)	Frequency (Hz)
0,121465965	2,4045887
0,12984293	3,2237096
0,13089005	3,239431
0,13403142	3,2878714
0,13633508	3,534844
0,13696335	3,4853284
0,13759163	3,745738
0,13905759	4,072459
0,1401047	4,514403
0,14136125	4,6628146
0,14638744	4,990678
0,15099477	6,2518096
0,284	1,3341058
0,284	1,3955034
0,284	1,255175
0,284	2,0745685
0,285	1,3054361
0,285	1,232577
0,0475	4,463618
0,0475	7,307391
0,0475	4,5487375
0,0475	8,455681
0,0475	5,4159994
0,0475	11,224499
0,0525	3,4325213
0,0525	4,0436425
0,0525	4,632447
0,0525	5,1361976
0,0525	5,7008247
0,0525	6,123365
0,0525	8,978365
0,0525	12,668651
0,0525	13,494266
0,06	3,548579
0,06	4,821226
0,06	4,538209
0,06	4,1600246
0,06	3,404691
0,06	3,638677
0,06	4,9839406
0,06	4,464351
0,06	3,9671054
0,06	5,572526
0,06	5,380434
0,06	5,095141
0,06	9,156379
0,06	13,61442
0,06	14,382374
0,06	16,54514
0,06	15,588922
1,25	0,29610467
1,25	0,3513436
1,25	0,45396066
1,25	0,49449143
1,14	0,71550053
1,14	0,79524857
1,14	0,8039628
1,14	0,88489765
1,14	0,8955921
1,14	0,9271286
1,14	0,9558049
1,14	1,1546383
0,82	0,7321889
0,82	0,77208626
0,82	0,8053363
0,82	0,8301721
0,82	0,80406946
0,82	0,8868179
0,82	0,8869179
0,82	0,88141733
0,82	0,9103202
0,82	0,95599157
0,82	1,016191
0,82	0,91586083
0,82	1,0079569
0,82	1,1028798
0,82	1,1892219
0,82	1,091992
0,82	1,0261786
0,82	1,0320259
0,82	1,2906655
2,25	0,67
0,98	1,12
2,25	0,95
0,93	1,25
0,97	1,13
2,25	0,78
0,163	1,9283819
0,163	1,8753315
0,163	1,8169761
0,163	2,061008
0,163	2,4907162
0,158	2,0733628
0,158	2,732807
0,158	2,994443
0,158	3,002107
0,158	2,9872236
0,149	1,8585341
0,149	1,9707527
0,149	2,3683465
0,149	2,6965046
0,179	1,7685242
0,179	1,6976821
0,179	1,9924833
0,179	2,497167
0,179	2,607586
0,171	2,0722423
0,171	2,231035
0,171	2,5012054
0,171	2,535799
0,171	2,8696737
9,5	0,2
1,6	0,6
2,98	0,15
1,7	0,73
2,5	0,4
0,57	1,17
1	1,03
3,57	0,51
6	0,21
1,3	0,89
0,55	1,20243
0,55	1,445
0,55	1,91303
1,83	3,7602863
1,83	2,653492
1,83	2,740953
1,83	1,459266
1,83	2,9058304
1,83	2,235893
1,83	2,9057362
1,83	2,2260902
1,83	2,6046891
1,83	2,313445
1,83	2,7357628
1,83	2,7311027
2,2	3,3198
2,2	3,21326
2,2	3,75913
2,2	4,50796
2,2	3,08235
2,2	3,14802
2,2	3
2,2	2,65444
2,2	2,86783
2,2	3,01712
2,2	2,69007
2,2	2,93396
2,58	1,6412456
2,58	3,0083916
2,58	1,6237762
2,58	2,160839
2,58	1,5062937
2,58	2,0223777
2,58	2,9076922
2,58	1,9132867
2,58	1,5902098
2,58	2,0097902
2,58	2,937063
2,58	2,1692307
2,58	2,3412588
2,58	2,353846
2,58	1,8881118
2,58	0,7090909
2,58	2,1188812
2,58	1,9762238
2,58	0,9902098
2,58	2,0685315
2,58	1,4601399
2,58	1,413986
2,58	2,488112
2,58	0,8727273
2,58	1,5944057
2,58	1,5986013
2,58	1,1034966
2,58	0,9188811
2,58	1,1328671
2,58	1,7412587
2,58	1,0993007
2,58	2,3286712
2,58	1,7370629
2,58	1,372028
2,58	2,1356642
2,58	2,2657342
2,58	1,650231
2,58	1,0405594
2,58	1,7041221
2,58	1,3048952
2,58	2,206993
2,58	2,2111888
2,58	2,7734265
2,58	2,3581522
2,58	1,2713287
2,58	1,6909091
2,58	1,2377622
2,58	2,1482518
2,58	2,1734266
2,58	2,73986
2,58	1,2167832
2,58	1,0951049
2,58	2,4713287
2,9	2,88728
2,9	2,88764
2,9	3,5644
2,9	3,7431
2,9	3,24503
2,9	3,40739
2,9	3,76764
2,9	3,40673
2,9	3,40801
3,55	1,5788959
3,55	1,7028568
3,55	1,1609179
3,55	0,88346213
3,55	0,6698162
3,55	0,7342817
3,55	1,2292589
3,55	1,5107481
3,55	1,5067089
3,55	1,5153437
3,55	0,88362604
3,55	1,0928696
3,55	0,9044843
3,55	0,7895797
3,55	0,6102798
3,55	1,3699099
3,55	0,6658705
3,55	1,4130195
3,55	1,4471198
3,75	2,5805454
3,75	1,5941509
3,75	1,5900755
3,75	1,5892043
3,75	1,5801365
3,75	1,39681
3,75	1,3931016
3,75	1,5270656
3,75	2,104111
3,75	1,3845726
3,75	1,5139111
3,75	1,1750115
3,75	2,325233
3,75	1,6816585
3,75	1,3729255
3,75	1,4766027
3,75	0,8483093
3,75	1,7111717
3,75	1,7904485
3,75	2,2387283
3,75	2,179748
3,75	2,9196684
3,75	1,8818824
3,75	2,6935482
3,75	1,9952178
3,75	1,9945643
3,75	1,782401
3,75	0,75205487
3,75	2,5676432
3,75	1,3117728
3,75	2,163779
3,75	2,1780226
3,75	2,0124705
3,75	2,1610909
3,75	2,1640427
3,75	3,2098877
3,75	2,311723
3,75	1,9927359
3,75	2,4868188
3,75	0,8276232
3,75	1,6767923
3,75	2,1727896
3,75	1,5141404
4,74	1,86378
4,74	2,316552
4,74	1,5020921
4,74	1,3470527
4,74	1,5355371
4,74	1,4856647
4,74	1,5527784
4,74	1,4733185
4,74	1,5738887
4,74	1,6827183
4,74	1,7624819
4,74	1,871965
4,74	1,5561602
4,74	1,620041
4,74	1,8548957
4,74	1,7577302
4,74	1,0735027
4,74	1,7583321
4,74	1,8719306
4,74	1,9884523
4,74	1,6660388
4,74	1,4857049
4,74	1,68657
4,74	1,5530019
4,74	1,4816697
4,74	1,4899808
4,74	1,7963912
4,74	1,8718848
4,74	1,507222
5,6	1,8631
5,6	2,32143
5,6	2
19	0,28
19	0,28
19	0,25
19	0,27
19	0,28
19	0,28
19	0,23
3,34	0,2602046
3,34	0,28046882
3,34	0,310056
3,34	0,3005551
3,34	0,34053957
3,34	0,34052825
3,34	0,34050104
3,34	0,33039388
3,34	0,31999773
3,34	0,3096016
3,34	0,32026178
3,34	0,29058734
3,34	0,29998738
3,34	0,309401
3,34	0,29988652
3,34	0,35969436
3,34	0,3694083
3,34	0,3403628
3,34	0,32990432
3,34	0,3203921
3,34	0,32037622
3,34	0,32004532
3,34	0,36014652
3,34	0,37083274
3,34	0,34972543
3,34	0,34026194
3,34	0,34938434
3,34	0,36003092
3,34	0,34013614
3,34	0,35019347
3,34	0,3602247
3,34	0,3293003
3,34	0,3399979
3,34	0,38967022
3,34	0,3787993
3,34	0,3983065
3,34	0,39859545
3,34	0,40978372
3,34	0,4690612
3,34	0,42965695
3,34	0,4299108
3,34	0,43965307
3,34	0,46912467
3,34	0,44898966
3,34	0,43946043
3,34	0,41931182
3,34	0,42928186
3,34	0,40909246
3,34	0,49989948
3,34	0,548604
5,60144	0,5137405
5,60144	0,47382703
5,62701	0,43287614
7,42934	0,37888935
7,42934	0,35447595
7,70506	0,22882535
8,21243	0,22882535
8,21243	0,22882535
8,99573	0,21104747
9,45795	0,20124218
9,89873	0,1965117
10,5026	0,20124218
10,36	0,22028007
12,4873	0,18649241
13,2491	0,18649241
13,0099	0,17200346
13,6162	0,16876125
1,03	0,77
1,09	1,09
1,03	1,14
1,09	1
1,09	1,2
1,03	1,14
1,03	1,26
1,64	0,55
1,64	0,71
1,43	0,8
1,43	0,92
1,43	1,14
4,50607691	0,538777933
4,526137466	0,441663256
5,734022596	0,401298097
5,743526114	0,493544708
5,934812463	0,452734155
5,947043292	0,447601445
6,505462833	0,426295747
6,506754184	0,382836203
6,51708732	0,488816571
6,569949631	0,50831298
7,002788341	0,360471846
7,041938859	0,482530978
7,047781641	0,307781492
7,056726733	0,467062855
7,145786561	0,39363097
7,757578876	0,278242675
7,831228572	0,415417933
7,90840551	0,510915173
7,947218434	0,327484568
[illegible]	[illegible]
